# The INTESTINE study: INtended TEmporary STomas In crohN’s diseasE. Protocol for an international multicentre study

**DOI:** 10.1007/s13304-022-01345-y

**Published:** 2022-08-12

**Authors:** Valerio Celentano, Claire Perrott, Patricia Tejedor, Giacomo Calini, Matteo Rottoli, Christos Kontovounisios, Paris Tekkis

**Affiliations:** 1 Chelsea and Westminster Hospital NHS Foundation Trust, London, UK; 2 Department of Surgery and Cancer, Imperial College, London, UK; 3 University Hospital Gregorio Marañon, Colorectal Surgery Unit, Madrid, Spain; 4 Clinica Chirurgica, University Hospital Udine, Udine, Italy; 5 Surgery of the Alimentary Tract, IRCCS Azienda Ospedaliero Universitaria di Bologna, Bologna, Italy; 6 Alma Mater, Studiorum University of Bologna, Bologna, Italy

**Keywords:** Crohn's disease, Inflammatory bowel disease, Ileostomy, Colostomy

## Abstract

Surgery for ileocolonic Crohn’s disease can result in temporary or permanent stoma formation which can be associated with morbidity as parastomal and incisional hernias, readmissions due to obstruction or high stoma output, and have a negative impact on quality of life. We propose an international retrospective trainee-led study of the outcomes of temporary stomas in patients with Crohn’s disease. We aim to evaluate both the short-term (6 month) and mid-term (18 month) outcomes of temporary stomas in patients with Crohn’s Disease. Retrospective, multicentre, observational study including all patients who underwent elective or emergency surgery for ileal, colonic and ileocolonic Crohn’s disease during a 4-year study period. Primary outcome is the proportion of patients who still have an ileostomy or colostomy 18 months after the initial surgery. Secondary outcomes: complications related to stoma formation and stoma reversal surgery; time interval between stoma formation and stoma reversal; risk factors for stoma formation and non-reversal of the stoma. We present the study protocol for a trainee-led, multicentre, observational study. Previous research has demonstrated significant heterogeneity surrounding the formation and the timing of reversal surgery in patients having a temporary ileostomy following colorectal cancer surgery, highlighting the need to address these same questions in Crohn’s disease, which is the aim of our research.

**Supplementary Information:**

The online version contains supplementary material available at 10.1007/s13304-022-01345-y.

## Background

Europe has one of the highest prevalence of inflammatory bowel disease (IBD) across the world [1]. In the UK 115,000 patients are affected by Crohn’s disease (CD) [2] and up to 80% of these patients will require surgical intervention at some point in their lives, despite medical treatment [3]. Indications for surgery include perianal disease, enteric fistulae, perforation, obstruction, strictures and medically refractory disease. Surgical resection can result in temporary or permanent stoma formation which can be associated with morbidity due to parastomal and incisional hernias, prolapse and have a negative impact on quality of life. The formation of stomas can cause prolonged and repeated hospital admissions due to high output or bowel obstruction, and other stoma-related complications.

Patients with ileocolonic CD often undergo multiple operations throughout their lives and so are exposed to these risks at each procedure. Key performance indicators (KPIs) can be used as a quality measurement to monitor standards of care across a variety of healthcare settings, and KPIs for the surgical management of Inflammatory Bowel Disease have been proposed by a Delphi Consensus study [4] including not only postoperative complications and rate of re-interventions and readmissions but also the proportion of patients who had a defunctioning stoma and if this was reversed in a timely fashion.

Currently, no study has investigated what happens to these temporary stomas in CD and whether planned reversals happen and if not, what are the reasons.

### Aims

We propose an international retrospective trainee-led snapshot study of the outcomes of temporary stomas in patients with Crohn’s disease. We aim to evaluate both the short-term (6 month) and mid-term (18 month) outcomes of intended temporary stomas in patients with Crohn’s Disease.

## Methods

### Study design

International, retrospective, multicentre, observational study.

### Study objectives

- To determine the proportion of temporary stomas reversed at 6- and 18-months post formation in patients with Crohn's disease.
- To determine the time interval between stoma formation and stoma reversal.
- To determine risk factors for the formation of temporary stoma and risk factors for delayed or no reversal.
- To establish the morbidity related to the presence of a stoma in patients with Crohn’s disease
- To inform the consenting process for patients undergoing Crohn’s Disease surgery, by acknowledging risk factors for delayed or no reversal.

### Study setting

International retrospective multicentre study was designed according to the SPIRIT guidelines [5]. Participating centres must hold a regular IBD MDT and have dedicated IBD colorectal surgeons and gastroenterologists. Participating Centres must provide a local lead and a local trainee lead. Study steering group includes patients’ representatives, surgical trainees, colorectal surgeons with expertise in CD surgery, methodological leads with expertise in leading and developing multicentre studies.

### Study participants

Patients who have had a stoma formation for Crohn’s Disease.

### Inclusion criteria

The participant must meet the following criteria to be considered eligible for inclusion in the study:

- Aged 18 years or over
- Underwent an intended temporary stoma formation for Crohn’s disease
- Between January 2017–December 2020, the 4-year study recruitment period.
- Follow-up of 18 months for each patient
- Either elective or urgent/emergency surgery
- The procedures included are:
  o ileocolonic resection (right hemicolectomy, extended right hemicolectomy, ileocaecal resection, redo ileocolic resection),
  o segmental colonic resections including subtotal colectomy, left hemicolectomy, transverse colectomy, anterior resection, Hartmann’s procedure,
  o small bowel resection, strictureplasty as a sole procedure,
  o formation of ileostomy or colostomy as a sole procedure for the treatment of complex ileocolic disease or perianal disease.

### Exclusion criteria

- Excluded procedures will be panproctocolectomy and proctectomy.

#### Primary outcome

The proportion of patients who still have an ileostomy or colostomy 18 months after the initial surgery.

#### Secondary outcomes

- Complications related to stoma formation and stoma reversal surgery.
- Time interval between stoma formation and stoma reversal
- Risk factors for stoma formation and non-reversal of the stoma.

#### Subgroup analysis

The cohort of patients having a stoma in the context of colonic CD or perianal CD will be separately evaluated for the primary and secondary outcomes.

#### Accessory evaluation

The impact of Covid on the reversal rate of stomas. To compare the rate and timing of stoma reversal pre and post the Covid pandemic, if there is overlap in the data.

#### Sample size

We aim to evaluate what happens to patients who had a stoma formed during their surgery for CD and therefore there is no need to limit the sample size to a specific number of patients being this is a snapshot study [6]. For the statistical analysis fn the group of patients having ileocaecal or redo ileocolonic resection, the minimum sample size was calculated considering an 11% incidence of new stoma formation in patients undergoing surgery for ileocolonic CD, according to previously published literature [7]. With a confidence level of 95% and error margin of 5%, 151 patients were required.

### Study procedures

First, the study was registered at Chelsea and Westminster Hospital. Subsequently, the study will be disseminated to IBD centres worldwide, to involve IBD consultant surgeons to act as lead consultants and trainee leads for each participating centre. We will use virtual meetings to communicate with centres and use email to circulate information on the methodology for data collection. The recruitment of new centres will continue during the data collection period. A national/region all study lead will be identified for each participating country to act as a bridge between each participating centre and the study steering group.

### Ethical considerations

All data collected will measure current practice, with no changes made to normal treatment. As such, this study should be registered as an audit of current practice at each participating centre. It is the responsibility of the local team at each site to ensure that local audit approval (or equivalent) is completed for their centre. Participating centres will be asked to confirm that they have gained formal approval at their site. When required, ethical approval will be sought by the individual participating centres according to local policies.

#### Data collection

We will allocate 12 weeks for data collection. Initially, each centre will review clinical records to identify patients who underwent Crohn’s disease surgery which involved the formation of an ileostomy or colostomy. Data will be collected on these patients including:

- *Demographic data:* Age, gender
- *Disease-specific information*: Crohn’s phenotype and Montreal classification [8], history of previous surgery, preoperative medical treatments, body mass index and preoperative weight loss. Harvey–Bradshaw disease activity score [9].
- *Surgery specific*: indication for surgery, surgery scheduling (elective, emergency) operation performed, surgical approach (laparoscopic, open, robotic), indication for the stoma including, the type of stoma created (ileostomy/colostomy) and if any plans for stoma reversal are stated.
- *Surgical outcomes*: the length of hospital stay, 30-day morbidity (according to Dindo-Clavien classification) [10], for both the index surgical procedure when the stoma was fashioned and for the stoma reversal surgery (if performed), long-term data on whether stoma had been reversed at 6 and 18 months and if any complications relating to the stoma occurred in the meantime.
- *Patient Reported Outcome Measures (PROMs):* Data on PROMs will be included if collected.

Sites will be expected to identify eligible patients from the established database, operating theatre registries, IBD MDT registries, and clinical coding searches.

### Statistical analysis

Categorical variables are presented as frequency and percentages, and compared using the chi-square test or Fisher’s exact test, as appropriate. Continuous variables are presented as mean (± standard deviation) or median (range) according to their distribution and compared with the use of Student’s t test or the Mann–Whitney *U* test in case of normal or skewed distribution, respectively. To identify variables associated with binary outcomes, uni- and multivariable logistic regression analyses will be performed. Variables having a *p* value equal to 0.10 or less at the univariate analysis will be included in the multiple regression model. The Odds ratio (ORs) with a 95% confidence interval (CI) was estimated as a measure of association. Statistical analysis will be performed using IBM SPSS Statistics for Windows, version 25.0 (IBM Corp., Armonk, NY, USA).

### Data storage

Data will be collected and stored on the Research Electronic Data Capture (REDCap) of the University of Bologna. No patient identifiable data (name, date of birth, address, etc.) will be recorded on REDCap. Registered local investigators will have individual password-protected access to their unit's data entered onto REDCap. During the running of the audit, only local data will be visible to investigators; other sites’ data will not be accessible.

### Timetable

- Recruitment and dissemination of study protocol: Month 1 to month 4.
- Data collection: Month 5 to month 8.
- Analysis: Month 9 to Month 10
- Writing up reports/publications/dissemination: Month 11 onwards.

### Dissemination plan

The findings will be disseminated using multiple routes, including:

- Relevant webpages, social media, events of Crohn’s disease, IBD and colorectal conditions charities.
- Locally at our annual research and Innovation conference.
- Presentation at National and International conferences.
- Published in peer-reviewed academic journals.
- Professional media and accessible formats presented to already established focus groups of patients.

### Authorship

Collaborative authorship will be offered to local leads and trainees at each centre for submissions to peer-reviewed journals.

## Discussion

The presence of a stoma can significantly affect patients’ quality of life [11, 12] and has also a risk of complications and re-interventions [13]. Stoma rates up to 35% have been described for complicated CD [14] and a penetrating phenotype of CD may explain a more common use of stomas in selected patients, because of abscesses and intra-abdominal contamination, or due to complex internal fistulae requiring more than one intestinal resection. Previous studies have reported a successful reversal rate in only 16.6% of patients having an intended temporary stoma for the treatment of perianal CD [15], demonstrating that any stoma fashioned in patients with CD must not be assumed as temporary.

Unfortunately, many key performance indicators of CD surgery such as postoperative morbidity, rate of ileostomy formation, reoperations and readmissions are not routinely recorded, with a paucity of audits on Patients Reported Outcome Measures (PROMs) [4].

Our study aims to fill this gap in the literature, and the collaborative nature of this multicentre study, will allow capturing real-world data on the current use of stomas in patients with CD and on the complications related to the stoma formation and reversal. One in five patients are readmitted with a stoma-related complication within 30 days of the creation of an ileostomy, with dehydration being the leading cause, occurring in 6% of patients [16]. The rate of stoma-related complications will likely be higher during the 18-months follow-up of our study, reflecting the high health care cost for a potentially avoidable cause, including long-term complications, which often require further surgery or contribute to mortality [11].

Previous studies in CD surgery have demonstrated significant variability across different hospitals in the volume of surgical procedures performed per year, the short-term surgical outcomes, and the adoption of minimally invasive surgery [17]. It is not surprising to highlight variations in IBD surgical practice, as similarly, the wide range of available treatments for IBD has been shown to result in significant heterogeneity amongst physicians in the use of biologics and combination therapy, confirming the need for standardised pathways for the care of IBD patients [18], and guidelines of several international societies have been released to optimize CD outcomes [19, 20]. The results of our study could contribute to the discussion on the centralisation of services for complex CD surgery [21], by obtaining real-world data on current practice in CD. We expect that our study will demonstrate divergence across different hospitals and countries in the proportion of CD patients having their temporary stomas reversed, and in the time gap between stoma formation and reversal, considering the potential higher risk of complications when this gap is longer.

We must acknowledge that not all stomas in patients with CD are intended as temporary, as for example in case of severe perianal involvement, or when there is an extensive multi-level disease in the bowel segments distal to the stoma, and the retrospective design of our study might not be able to capture these exemptions, similarly to stomas not reversed as per patients’ preference. Nevertheless, awareness of the proportion of CD patients who successfully have their stoma reversed in a timely manner could forge counselling and decision making, as for example in patients with complicated colonic diverticulitis who have a Hartmann’s procedure, which is never reversed up to 40% of the patients [22]. Previous research has demonstrated the significant heterogeneity surrounding the formation, and more importantly, the timing of reversal surgery in patients having a temporary ileostomy following anterior resection for cancer [23], highlighting the need to address these same questions in CD surgery as well.

## Supplementary Information

Below is the link to the electronic supplementary material.

Appendix 1 Study steering group. Supplementary file1 (DOCX 12 KB)

Appendix 2 SPIRIT checklist. Supplementary file2 (DOC 124 KB)

Appendix 3 Proposed CRF. Supplementary file3 (PDF 110 KB)
