# Supplementary material for: The INTESTINE study: INtended TEmporary STomas In crohN’s diseasE. Protocol for an international multicentre study
Source: Updates Surg. 2022 Aug 12;74(5):1691–6. doi: 10.1007/s13304-022-01345-y (PMC9481503; doi:10.1007/s13304-022-01345-y)
Supplement: Supplementary file 1 — Study steering group. Supplementary file1 (DOCX 12 KB) [file 13304_2022_1345_MOESM1_ESM.docx]

**Appendix 1. Study Steering Group and National Leads**

Principal Investigator: Mr Valerio Celentano

Associate Principal Investigator: Dr Claire Perrott

Associate Principal Investigator: Prof Matteo Rottoli

Methodological Lead: Mr Christos Kontovounisios

Patient representative: Ms Sue Blackwell

National coordinators: Ms Patricia Tejedor, Dr Vincenzo Vigorita, Dr Orsalia Mangana, Prof Nicola de’ Angelis, Dr Giacomo Calini, Muhammed El-Hadi
