# Supplementary material for: The INTESTINE study: INtended TEmporary STomas In crohN’s diseasE. Protocol for an international multicentre study
Source: Updates Surg. 2022 Aug 12;74(5):1691–6. doi: 10.1007/s13304-022-01345-y (PMC9481503; doi:10.1007/s13304-022-01345-y)
Supplement: Supplementary file 3 — Proposed CRF. Supplementary file3 (PDF 110 KB) [file 13304_2022_1345_MOESM3_ESM.pdf]

INTESTINE

INtended TEmporary STomas In crohN's diseasE

REDCap Unique ID:

SECTION 1: Patient Information

Age at surgery	_____yrs	Gender	Male Female	Smoking	Yes No	Previous abdominal surgery	Yes No
Disease activity index				Perianal disease	Yes No	If previous abdominal surgery, please specify	
Montreal Classification	A<br>L<br>B			Medical treatment prior to index surgery, please specify:			
Crohn's Disease presentation		Primary	Recurrent				

SECTION 2: Index Operation

Date of surgery		Date of stoma formation		ASA grade	
Indication for surgery	☐ Abscess ☐ Perforation ☐ Failed medical treatment<br>☐ Fistula ☐ Stricture ☐ Other	If other, please specify			
Main procedure:	☐ Ileocaecal resection/right hemicolectomy<br>☐ Re-do Ileocolic resection<br>☐ Small Bowel resection<br>☐ Strictureplasty as sole procedure<br>☐ Subtotal colectomy<br>☐ Segments colectomy (Left Hemicolectomy, anterior resection, transverse colectomy<br>☐ Laparoscopic assisted formation of stoma (sole procedure)	Pre-Op review by stoma nurse:	Yes No	Timing of surgery	☐ Elective<br>☐ Urgent<br>☐ Emergency
		Associated procedure:	Yes No		
		If Yes, Associated procedure:	☐ Ileocaecal resection/right hemicolectomy<br>☐ Redo ileocolic resection<br>☐ Small bowel resection<br>☐ Strictureplasty<br>☐ Segmental colectomy (left hemicolectomy, anterior resection, transverse colectomy)	Surgical Approach	☐ Open<br>☐ Laparoscopic<br>☐ Laparoscopic converted to open<br>☐ SILS<br>☐ SILS converted to conventional laparoscopy<br>☐ SILS converted to open<br>☐ Robotic<br>☐ Robotic converted to open
Was the stoma formed during the index operation or made as a result of postoperative complication?	☐ Complication<br>☐ Index procedure	If due to a complication, please specify		Type of stoma formed	☐ End Ileostomy<br>☐ Loop ileostomy<br>☐ End Colostomy<br>☐ Loop Colostomy
What type of anastomosis was fashioned:		Was an anastomosis fashioned at the time of index stoma formation			Yes No
		How was the distal end managed?		☐ Mucus fistula<br>☐ Closed & left inside abdominal cavity	

Intraoperative Data

Blood Loss		Any intraoperative complications?	Yes No
Operating time	_________ minutes	Please specify complications	

Post-operative course

Length of stay:	____ days	Complications	Yes No	Intra-abdominal collection		Yes No	
Anastomotic Leak	Yes No	Wound Infection	Yes No	DVT	Yes No	PE	Yes No
Ileus	Yes No		Stoma prolapse:	Yes No	High stoma output:		Yes No
Re-operation required	Yes No		Details of reoperation:		Refashioning of stoma required		Yes No
Re-admission:	Yes No		Details of re-admission:				

Follow Up

At 6 months follow up has the stoma been reversed	Yes No	Was the patient readmitted in the 18 months after surgery for any of the following:	☐ High output stoma ☐ Refashioning of stoma<br>☐ Bowel obstruction<br>☐ Stoma prolapse ☐ Stoma stenosis
At 18 months follow up has the stoma been reversed	Yes No		
If readmission, please specify how many times and what treatment was required			

SECTION 3: Stoma Reversal Surgery

Date of surgery:		Procedure performed:	☐ Formation of small bowel anastomosis<br>☐ Formation of ileocolic anastomosis<br>☐ Formation of colonic anastomosis	Approach	☐ Peristomal incision only<br>☐ Laparotomy<br>☐ Laparoscopy
Associated procedure		Yes No			
Associated procedures if yes:	☐ Ileocaecal resection/right hemicolectomy<br>☐ Redo ileocolic resection	☐ Small bowel resection<br>☐ Strictureplasty<br>☐ Segmental colectomy (left hemicolectomy, anterior resection, transverse colectomy)			

Intraoperative Data

Blood Loss		Any intraoperative complications?	Yes No
Operating time		Please specify	

Post-operative course

Length of stay		Complications	Yes No	Intra-abdominal collection		Yes No	
Anastomotic Leak	Yes No	Wound infection	Yes No	DVT	Yes No	PE	Yes No
Ileus	Yes No		Stoma prolapse	Yes No	High stoma output	Yes No	
Re-operation	Yes No		Details of reoperation		Refashioning of stoma	Yes No	
Re-admission:	Yes No		Re-admission, please specify				
